# Prognostic value of Dicer expression in human breast cancers and association with the mesenchymal phenotype

**DOI:** 10.1038/sj.bjc.6605193

**Published:** 2009-08-11

**Authors:** G Grelier, N Voirin, A-S Ay, D G Cox, S Chabaud, I Treilleux, S Léon-Goddard, R Rimokh, I Mikaelian, C Venoux, A Puisieux, C Lasset, C Moyret-Lalle

**Affiliations:** 1 Université de Lyon, Université Lyon 1, ISPB, Lyon, F-69003, France; 2 Inserm, U590, Lyon, F-69008, France; 3 Centre Léon Bérard, Lyon, F-69008, France; 4 Université de Lyon, Université Lyon 1, Faculté Grange Blanche, CNRS, UMR 5558, Laboratoire de Biométrie et Biologie Evolutive, Lyon, F-69373, France; 5 Hospices Civils de Lyon, Hôpital Edouard Herriot, Service d’Hygiène, Epidémiologie et Prévention, Lyon, F-69437, France; 6 Centre Léon Bérard, Département de Santé Publique, Lyon, F-69008, France; 7 Centre Léon Bérard, Service d’Anatomopathologie, Lyon, F-69008, France; 8 Université de Lyon, université Lyon 1, Faculté Grange Blanche, CNRS, UMR5201, Laboratoire de Génétique Moléculaire, Signalisation et Cancer, Lyon, F-69008, France

**Keywords:** Dicer, mRNA, quantitative RT–PCR, TMA, prognostic value, breast cancer

## Abstract

**Background:**

Dicer, a ribonuclease, is the key enzyme required for the biogenesis of microRNAs and small interfering RNAs and is essential for both mammalian development and cell differentiation. Recent evidence indicates that Dicer may also be involved in tumourigenesis. However, no studies have examined the clinical significance of Dicer at both the RNA and the protein levels in breast cancer.

**Methods:**

In this study, the biological and prognostic value of Dicer expression was assessed in breast cancer cell lines, breast cancer progression cellular models, and in two well-characterised sets of breast carcinoma samples obtained from patients with long-term follow-up using tissue microarrays and quantitative reverse transcription–PCR.

**Results:**

We have found that Dicer protein expression is significantly associated with hormone receptor status and cancer subtype in breast tumours (ER *P*=0.008; PR *P*=0.019; cancer subtype *P*=0.023, luminal A *P*=0.0174). Dicer mRNA expression appeared to have an independent prognostic impact in metastatic disease (hazard ratio=3.36, *P*=0.0032). In the breast cancer cell lines, lower Dicer expression was found in cells harbouring a mesenchymal phenotype and in metastatic bone derivatives of a breast cancer cell line. These findings suggest that the downregulation of Dicer expression may be related to the metastatic spread of tumours.

**Conclusion:**

Assessment of Dicer expression may facilitate prediction of distant metastases for patients suffering from breast cancer.

Breast cancer is the leading cancer diagnosis among women in the western world. With ever-improving chemotherapeutic, radiation, hormonal treatments, as well as HER2 and EGFR antagonists, an improvement in overall survival has been observed. However, the treatment of breast cancer is currently far from being optimal with patients suffering from recurrent breast carcinoma usually dying of the disease. Metastasis represents one of the final stages of the potentially lethal evolution of breast cancer. The natural progression of breast cancer differs greatly between patients, and metastatic progression in breast cancer is a complex and largely unknown process. The different biological behaviours observed among the distinct breast cancer subtypes may suggest different mechanisms of invasion and metastasis for breast tumours. Current therapy decision making is increasingly governed by the molecular classification of breast cancer (luminal A, luminal B, basal like, HER2+, hormone receptor status). Cancer subtypes have characteristic sites to which they metastasise (Luck *et al*, 2008). A very common metastatic site for human breast cancer is the bone. Genes involved in metastasis but not in primary tumourigenicity have been identified, particularly in bone metastases (Mundy, 2002). Identification of biological factors in relation to metastatic evolution and patterns is important for the optimal management of patients. We hypothesised that the expression of *Dicer* could be one such marker of prognosis in breast cancer.

The *Dicer* gene encodes a protein that functions as an RNase endonuclease type III and is required for the RNA interference and microRNA (miRNA) pathways. It produces miRNAs and small interfering RNAs (siRNAs) from pre-miRNAs and dsRNA, respectively. *Dicer* null mice are embryonically lethal with the depletion of stem cells (Bernstein *et al*, 2003). Furthermore, embryonic stem cells that lack *Dicer* are viable but incapable of differentiation and show severe proliferation defects (Murchison *et al*, 2005). Dicer has been shown to be essential in stem cell maintenance by its involvement in auto-renewal and proliferation (Jin and Xie, 2007).

Owing to its central role in the post-transcriptional regulation of miRNA, Dicer is increasingly evoked in cancer-related studies. miRNAs have been shown to be differentially expressed between normal and malignant tissues (Volinia *et al*, 2006; Zhang *et al.*, 2006). Clearly, distinct miRNA expression signatures are found in different types of cancer (Calin *et al*, 2005; Iorio *et al*, 2005; Lu *et al*, 2005; Blenkiron *et al*, 2007). As genomic changes and transcriptional regulation of miRNA expression do not explain the differences in miRNA profiles between normal and malignant tissues, the deregulation of miRNA biogenesis was investigated in some cancers (Zhang *et al*, 2006; Blenkiron *et al*, 2007). Therefore, we asked whether the expression levels *of Dicer* could vary during breast cancer progression both at the transcriptional and post-transcriptional levels, as has been shown in ovarian, lung, and prostate cancers (Chiosea *et al*, 2006, 2007; Flavin *et al*, 2008; Merritt *et al*, 2008). We aim at answering this question using real-time reverse transcription (RT)–PCR, tissue microarray (TMA), and western blotting. In this study, we report an alteration in Dicer expression that could be an independent prognostic factor for metastatic evolution of breast tumours.

## Materials and methods

### Tumour samples

Frozen tissue samples were used for mRNA analyses, and paraffin-embedded tissue blocks were used for immunohistochemical analyses.

#### mRNA study

Tissue specimens were obtained from Eric Tabone (Biological Resources Department, Centre Léon Bérard, Lyon, France, French agreement no. DC-2008–99) and were collected before the initiation of any therapy from 104 patients suffering from breast cancer diagnosed between 1992 and 1999, who underwent surgery at the Centre Léon Bérard. Normal breast tissue samples were also obtained from four individuals (healthy individuals undergoing reduction surgery).

#### Immunohistochemical analysis

Formalin-fixed paraffin-embedded breast tumour samples obtained from 86 Centre Léon Bérard (French agreement no. DC-2008–99) patients diagnosed in 1998 with invasive breast cancer were used. Normal breast tissue samples were also obtained from eight individuals (four healthy individuals undergoing reduction surgery and four breast cancer patients with normal surrounding tissues). None of the participants evaluated in the mRNA study were present in the immunohistochemical analyses. All human tissue samples were collected after obtaining approval from the Comité de Protection des Personnes Lyon Est and by the institutional review board and ethics committee of Centre Léon Bérard, with fully informed patient consent.

### Cellular models and cell lines

Human mammary epithelial cells (HMECs)-hTERT, HMECs-hTERT+ LT, and HMLER (cells expressing hTERT, LT, and H-rasV12) were first derived and kindly provided by RA Weinberg and M Brooks (Whitehead Institute for Biomedical Research, Ludwig Center for Molecular Oncology, MIT Department of Biology, Cambridge, MA, USA). The BO2 bone derivative MDA-MB-231 clone was kindly provided by P Clezardin (Research Unit U664, Laennec School of Medicine, INSERM, Lyon, France), and the single cell-derived progeny 2 (SCP2) bone derivative MDA-MB-231 clone was kindly provided by Yibin Kang (Department of Molecular Biology, Princeton University, Princeton, NJ, USA) and Joan Massague (Cancer Biology and Genetics Program, and Howard Hughes Medical Institute, Memorial Sloan-Kettering Cancer Centre, NY, USA). The 67NR, 168 FARN, 4TO7, 66c14, and 4T1 cell lines were first derived and kindly provided by F Miller (Karmanos Cancer Centre, Detroit, MI, USA) (Miller *et al*, 1983). The human mammary HMECs-hTERT, HMECs-hTERT+ LT, and HMLER cell lines, the SCP2 and BO2 bone derivatives, and the mouse mammary tumour cell lines (67NR, 168 FARN, 4TO7, 66c14, and 4T1) were maintained as described (Aslakson and Miller, 1992; Elenbaas *et al*, 2001; Minn *et al*, 2005; Garcia *et al*, 2008). A total of 21 breast cancer cell lines were obtained from the American Type Culture (http://www.ATCC.org) (CGC Standard Sonl, Mobheim, France) (see Supplementary Materials and Methods).

### RNA extraction and real-time PCR

Total RNA extraction was performed with a phenol–chloroform method, using TriReagent (Sigma-Aldrich, Saint Louis, MO, USA) for cell lysis and PhaseLockGel tubes (Eppendorf, Hamburg, Germany) for phase separation. After DNAse treatment, the DNA contamination of each sample was checked by electrophoresis on agarose gel. The synthesis of cDNA was performed using the First-Strand cDNA Synthesis Kit (GE Healthcare, Chalfont St Giles, UK). Transcription of the *Dicer* gene produces 14 different mRNAs, 11 alternatively spliced variants and 3 full-length forms (which vary in their 3′-untranslated region (3′UTR)). We chose primer sets to amplify the three a, b, and c full-length isoforms (http://www.ncbi.nlm.nih.gov/ieb/research/acembly/). Primer sequences and real-time PCR experimental procedures using the LightCycler system (Roche Applied Science, Basel, Switzerland) are described in Supplementary Materials and Methods.

For miRNA analysis, real-time PCR was carried out as mentioned above, using TaqMan miRNA assays according to the manufacturer's instructions (Applied Biosystems, Foster city, CA, USA) on an ABI Prism 7000. All miRNA data are expressed relative to RNU44, a small nucleolar (sn) RNA TaqMan PCR performed on the same sample. Expression variations were calculated using the ΔΔCt method. The Taqman miRNA reverse transcription kit (hsa-miR-21: 4373090, has-miR-182: 4373271, has-miR-221: 4373077, sno202: 4380917, RNU44: 4373384) was used to amplify the different miRNAs.

### Immunohistochemistry

Formalin-fixed paraffin-embedded breast tumour samples were inserted in triplicate using a 600-*μ*m needle (Alphelys, Plaisir, France) in three TMA blocks. All normal tissue samples were analysed on full tissue sections. These blocks were subsequently cut into 4 *μ*m-thick slices. After deparaffinisation and rehydratation, endogenous peroxidases were blocked by incubating the slides in 5% hydrogen peroxide in sterile water. For heat-induced antigen retrieval, tissue sections were boiled for 40 min in 10 mM citrate buffer (pH 6) in a water bath. Non-specific binding was blocked with a protein-blocking reagent (Immunotech, Marseille, France) for 5 min. Slides were then incubated overnight at 4°C with a mouse monoclonal anti-Dicer antibody (clone 13D6, Abcam, Cambridge, UK) diluted at 1 : 150 using an antibody diluent solution (Chem Mate, Dako, Trappes, France). After rinsing in phosphate-buffered saline, the slides were incubated with a biotinylated secondary antibody bound to a streptavidin-peroxidase conjugate (Vectastain Elite ABC reagent, Vector, Abcys, Paris, France). The bound antibody was shown by adding the substrate, 3,3′-diamino benzidine (DAB substrate kit for peroxidase, Vector). Sections were counterstained with haematoxylin. After dehydration and mounting, they were analysed independently by both a pathologist and a technician. Both the intensity of cytoplasmic staining (3 grades) and the percentage of positive tumour cells were assessed. The intensity of staining was graded on a scale from 0 to 2: ‘0’ reflected a lack of immunoreactivity, ‘1’ reflected weak immunoreactivity, and ‘2’ reflected strong immunoreactivity. Cases of discordance were reviewed by both the investigators to reach a consensus. The investigators were unaware of the RT–PCR results while scoring staining.

### siRNA and transfection

The sequences (designed by Eurogentech, Liege, Belgium) of the siRNAs used in this study are as follows: si1Dcr 5′-AGAGGUACUUAGGAAAUUU-3′ (recognising *Dicer* variants a, b, and c) and the corresponding siCt1 5′-UUACCUCCUUAGAUACAAU-3′; si2Dcr 5′-GGAGCUUGAUUUGCAUGAU-3′ (recognising *Dicer* variants a, b, and c), and the corresponding siCt2 5′-CCAAACACGGCUUUCAAAU-3′. Transfection of HeLa cells was performed using jetSI-endo (Polyplus Transfection, Illkirch France), as described by the manufacturer. Cells were plated at 2.5 × 10^5^ and transfected with 50 nM of Dicer targeting siRNA or the corresponding non-targeting siRNA twice at 24-h intervals. The cells were harvested 48 h after the second transfection, and gene silencing was assessed using quantitative RT–PCR and western blot.

### Immunoblot detection of Dicer

**Figure 1:**
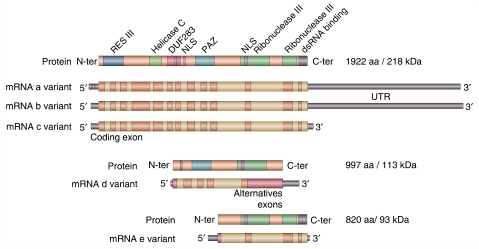
Schematic representation of five Dicer mRNA variants and their corresponding proteins according to AceView (http://www.ncbi.nlm.nih.gov/ieb/research/acembly/), transcription from the *Dicer* gene produces 14 mRNAs, 11 alternatively spliced variants and 3 full-length forms. The three full-length mRNA variants a, b, and c, the corresponding full-length protein (218 kDa) and the two alternatively spliced variants, d and e, and their corresponding proteins (113 and 93 kDa, respectively) are shown. Coloured areas represent the coding region and black bars represent 5′UTR and 3′UTR. Domain structures are represented on proteins.

Cell pellets were lysed with TEB150 buffer (50 mM HEPES; 150 mM NaCl; 2 mM MgCl_2_; 5 mM EGTA; 1 mM DTT; 0.5% Triton X-100; 10% glycerol; 1 mM Na_3_VO_4_; 10 *μ*l PIC-1 P2850, , and 10 *μ*l PIC-2 P5726, obtained from Sigma-Aldrich) for 30 min on ice. Western blot analyses were carried out as described previously (Wang *et al*, 2003) using a nitrocellulose membrane (BioRad, Marnes-la-Coquette, France). Membranes were incubated overnight with a 1 : 1000 dilution of human anti-Dicer antibody (clone 13D6, Abcam), or with a 1 : 750 dilution of mouse anti-Dicer antibody (Ab 13502, Abcam) overnight, followed by an incubation with 1 : 3000 anti-mouse IgG (Dako P0260) for 1 h at room temperature. Dicer expression was detected using the ECL western blotting detection and analysis system (GE Healthcare) according to the manufacturer's protocol. Relative protein expression was estimated using Quantity One software (BioRad). The monoclonal antibody used in this study for human Dicer recognises an epitope present on full-length protein (218.7 kDa) encoded by the three full-length transcripts a, b, and c and present on alternative proteins encoding by the d and e alternative transcripts (113.2 and 93 kDa, respectively) (Figure 1). To compare Dicer mRNA and protein levels, we quantified only the band corresponding to the known molecular weight of the Dicer protein (218.7 kDa).

### Identification of epithelial and mesenchymal phenotypes

Epithelial and mesenchymal phenotypes were determined in the 21 breast cancer lines and in the human mammary tumour progression cellular model by western blotting examining the expression of E-cadherin (epithelial marker) and vimentin (mesenchymal marker). Western blot analyses were carried out as described previously (Wang *et al*, 2003). Mouse monoclonal antibodies directed against E-cadherin (clone 36, Becton Dickinson, Glostrup, Denmark) and mouse monoclonal antibodies directed against vimentin (clone V9, Dako) were used. Epithelial and mesenchymal phenotypes were also determined by examining the morphology of the cells using an inverted microscope (Axiovert 25, Zeiss, Oberkochen, Germany).

### Clinical database description

Explanatory variables were extracted from the Centre Léon Bérard institutional breast cancer database, including those of all patients who underwent initial surgery performed at the institution since 1996. The database has been declared to the French authorities (CNIL). Parameters of interest include the clinical or surgical history of the patient, the histology of the tumour, the treatments applied, and immunohistochemical covariates (hormonal status, HER2). This breast cancer database was updated regularly for the follow-up data until the patient's death was reported (letter to the referring physician or registrar's office). The information on the evolution of the tumour in terms of local or distant recurrences is prospectively registered.

Clinical parameters taken into account in this study were:

- clinical data (age, menopausal status, metastatic status at diagnosis);
- histological data (histological type, pT (tumour size), metastatic lymph nodes status, SBR grade);
- immunohistochemical data (hormonal status, HER2 if available);
- cancer subtype (tumours were classified as luminal A, luminal B, HER2+, and basal like as described previously (Creighton *et al*, 2009; Hugh *et al*, 2009)).

### Statistical analysis

#### Immunohistochemistry expression and RNA expression

A comparison of the distribution of clinical parameters according to the intensity of Dicer cytoplasmic staining (0/1 *vs* 2) was made using Pearson's *χ*^2^ test; Fisher's exact test was used when the number of patients was small. For each cell line and tumour sample, repeated measurements of mRNA expression were obtained by replicating the extraction and reverse transcription steps. The mean mRNA expression and its 95% confidence interval were estimated using a hierarchical model (Sullivan *et al*, 1999; Goldstein, 2003). In these models, the mean mRNA expression of each cell line or tumour was allowed to vary randomly and to deviate from the group average according to within- and between-cell lines or tumour variances. This method adjusted for the within-cell line or tumour correlation from repeated observations, as well as estimated the mean of mRNA expression of each tumour. The median mRNA expression value was used to calculate a threshold separating low (⩽8) and high (>8) Dicer mRNA expressions among tumours.

#### Survival analysis

The end point of interest was metastasis-free survival defined as the time from the date of first diagnosis to the date of distant metastasis diagnosis or to the date of last follow-up for patients with no distant metastasis (censored observation). Survival analysis was carried out among the subgroup of patients with no metastasis at diagnosis. Survival estimates were calculated using the Kaplan–Meier method (Kaplan and Meier, 1958). The difference in survival estimates between the groups Dicer relative cDNA quantity ⩽8 and >8 was assessed using the log-rank test (Peto *et al*, 1977), and hazard ratios (HRs) were calculated using a Cox model.

Statistical analysis was carried out using SAS software version 9.1 (SAS OnlineDoc, Version 9, SAS Institute Inc., Cary, NC, USA) and R (R Development Core Team (2007). R: a language and environment for statistical computing R Foundation for Statistical Computing, Vienna, Austria. ISBN 3-900051-07-0, URL http://www.R-project.org) using the ‘nlme’ package (Pinheiro J, Bates D, DebRoy S and Sarkar D and the R Core team (2007). nlme: Linear and Nonlinear Mixed Effects Models. R package version 3.1–89).

## Results

### Dicer expression in tumour and metastatic progression cellular models and in breast cancer cell lines: downregulation in the more advanced stages and in mesenchymal phenotype breast cancer cell lines

**Figure 2:**
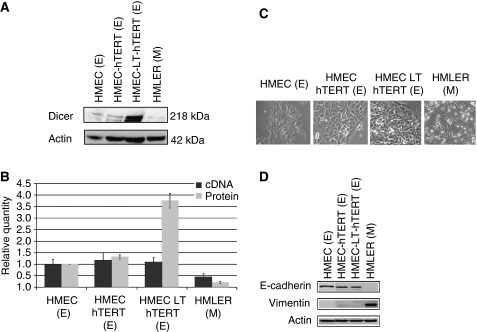
Expression of Dicer and EMT markers in a human mammary cancer progression cellular model. *Expression of Dicer in the human breast tumour progression cellular model*: (**A**) western blot analysis of Dicer expression in the four human breast cell lines, namely HMEC, HMEC+hTERT, HMEC+LT+hTERT, and HMLER (expressing H-rasV12) cells. The signal intensity of Dicer was normalised to that of actin. (**B**)The relative levels of Dicer mRNA were measured by real-time RT–PCR, and each bar represents the mean±s.d. of the PCRs in triplicate (▪). Dicer protein levels were quantified using Quantity One software (BioRad, Marnes-la-Coquette, France) and expressed as protein relative quantity. The ratios of Dicer/actin of three independent studies were expressed as mean±s.d. (
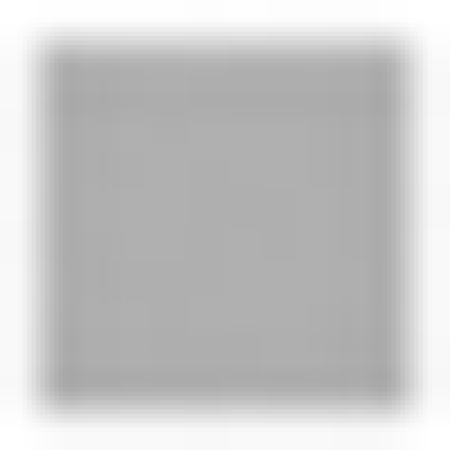
). *Morphological characteristics and expression of epithelial and mesenchymal markers in the human breast tumour progression cellular model*: (**C**) Morphological characteristics of the HMEC+hTERT, HMEC+LT+hTERT, and HMLER cells *in vitro* as assessed by phase-contrast microscopy. Images are shown at × 40 magnification. (**D**) Western blot analysis of E-cadherin and vimentin expression in the four human breast cell lines, HMEC, HMEC+hTERT, HMEC+LT+hTERT, and HMLER. Actin served as a loading control. E, epithelial phenotype; M, mesenchymal phenotype.

We assessed whether Dicer expression could be regulated transcriptionally and post-transcriptionally in the human cancer progression model developed by Elenbaas *et al* (2001) consisting of normal HMECs, HMECs+hTERT (telomerase catalytic subunit), HMECs+LT (SV40 large-T antigen)+hTERT, and HMLER (HMECs+LT+hTERT+H-rasV12). This model is believed to resume breast cancer progression. Dicer expression was readily detectable at both the mRNA and the protein levels in HMECs, HMECs+hTERT, and HMEC+LT+hTERT and was significantly decreased in HMLER (Figures 2A and B). Loss of E-cadherin (epithelial marker) and expression of vimentin (mesenchymal marker) are hallmarks of epithelial–mesenchymal transition (EMT). In this study, we examined the expression of E-cadherin and vimentin by western blot in HMECs and the in the three HMEC-derived cell lines. The morphological characteristics of these cell lines were assessed using phase-contrast microscopy (Figure 2C). Interestingly, we have shown that the decrease in Dicer expression is associated with EMT. Dicer levels were lower in the mesenchymal phenotype cell line, HMLER (Figure 2). HMLER cells have been shown previously to be a mixed population of mesenchymal and epithelial phenotype cells (Morel *et al*, 2008). In this study, the majority of the population of HMLER cells analysed were of the mesenchymal phenotype (Figures 2C and D). We analysed Dicer protein expression in hTERT+LT-RAS with either epithelial or mesenchymal phenotypes, and found that Dicer levels in the mesenchymal phenotype cells were lower than those observed in epithelial phenotype cells (data not shown). Given that the actin cytoskeleton is deeply reorganised during EMT (Zhang *et al*, 2009), we decided to compare the relative abundance of Dicer protein to actin with the relative abundance of Dicer protein to GAPDH by western blot. Using GAPDH as the protein-loading control, we found the same decrease in Dicer expression in HMLER cells compared with that in the other cell lines (Supplementary Figure S1). The expression of Dicer protein in epithelial phenotype cells relative to GAPDH was in the order of five- to 10-fold more abundant than in mesenchymal phenotype cells.

**Figure 3:**
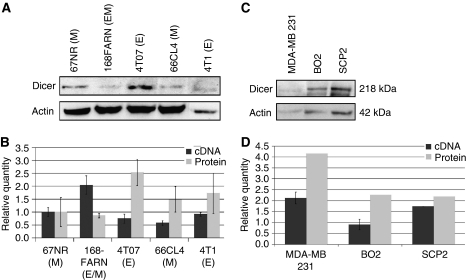
Expression of Dicer in metastasis progression cellular model and in bone metastatic cells. *Expression of Dicer in the mouse metastasis progression cellular model*: (**A**) western blot analysis of Dicer expression in the five mouse mammary cell lines, namely 67NR, 168FARN, 4TO7, 66c14, and 4T1. The signal intensity of Dicer was normalised to that of actin. (**B**) The relative levels of Dicer mRNA were measured by real-time RT–PCR, and each bar represents the mean±s.d. of the PCRs in triplicate (▪). Dicer protein levels were quantified using Quantity One software (BioRad, Marnes-la-Coquette, France) and expressed as protein relative quantity. The ratios of Dicer/actin of three independent studies were expressed as mean±s.d. (
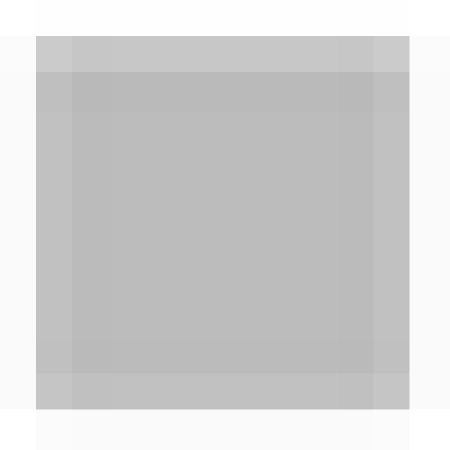
). E, epithelial phenotype; M mesenchymal phenotype; E/M, mixed phenotype. *Expression of Dicer in two bone metastatic derivatives of MDA-MB-231 cells*: (**C**) western blot analysis of Dicer expression in the three cell lines, namely MDA-MB-231, BO2, and SCP2. The signal intensity of Dicer was normalised to that of actin. (**D**) The relative levels of Dicer mRNA were measured by real-time RT–PCR, and each bar represents the mean±s.d. of the PCRs in triplicate (▪). Dicer protein levels were quantified using Quantity One software (BioRad, Marnes-la-Coquette, France) and expressed as protein relative quantity. The ratios of Dicer/actin of three independent studies were expressed as mean±s.d. (
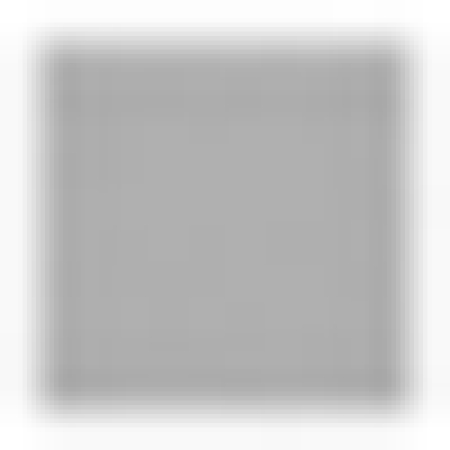
).

As the impact of a mesenchymal phenotype on the development and spread of tumours is poorly understood, we asked whether the downregulation of Dicer could be linked to tumour invasiveness. As no human model for metastasis progression in mammary tumour cells is available, we next analysed Dicer expression in a mouse model of invasiveness (Aslakson and Miller, 1992). This model consisted of five clonal tumour sub-lines derived from a spontaneously arising mammary tumour in a BALB/c mouse. The 67NR sub-line was considered as non-metastatic, 168 FARN and 4TO7 sub-lines formed micrometastases, whereas the 66c14 and T1 derivatives readily metastasised with differential organ specificities (Aslakson and Miller, 1992; Tester *et al*, 2001). They can be classified as following from the less tumourigenic to the highest 67NR<168FARN<4TO7<66c14<4T1. The behaviour of these tumour lines is believed to reflect the sequence of multi-step metastasis progression. Therefore, we set out to compare the expression profiles of Dicer mRNA and protein in these five sub-lines to investigate whether Dicer could be differentially regulated during metastasis progression. Dicer mRNA and protein levels showed some discrepancies among the five sub-lines, and no association was found between Dicer expression and capacities of the cells to metastasise *in vivo* (Figures 3A and B). However, we have found a correlation between Dicer protein levels and epithelial–mesenchymal phenotype in these sub-lines. The morphological characteristics of the five sub-lines were assessed using phase-contrast microscopy (data not shown).

After lymph node invasion, breast cancer commonly causes osteolytic metastases in the bone. We next addressed the issue of whether Dicer could be downregulated in a bone metastasis model of breast cancer cells. Mammary tumours do not spontaneously metastasise to the bone in mice, as is the case in humans, which hamper studies in animal models. We assessed Dicer expression in two distinct bone metastasis subpopulations both derived from the human breast cancer cell line MDA-MB-231. BO2 cells were obtained after injection of MDA-MB-231 cells into the tail vein (Bellahcène *et al*, 2007), and SCP2 cells were obtained after an intra-cardiac injection of parental cells into immune-deficient mice (Minn *et al*, 2005). Interestingly, we have found a significantly decreased expression of Dicer both at the mRNA and the protein levels in the BO2 and SCP2 cells in comparison with the parental cells (Figures 3C and D).

Inactivation of Dicer is associated with impaired maturation of miRNA (Cummins *et al*, 2006; Kumar *et al*, 2007; Wiesen and Tomasi, 2009) We have studied the expression of three mature miRNAs, namely miR-182, miR-221, and miR-21 that were involved in the tumourigenesis in Dicer knocked-down human tumour cells (Supplementary Figures S2A and B). The siRNA directed against Dicer were efficient against the three isoforms (a, b, and c) that coded for the full-length protein (Figure 1). In Dicer knocked-down cells as compared with controlled siRNA-transfected cells, we observed a global decrease in the three mature miRNAs (Supplementary Figure S2B). However, despite a strong inhibition of Dicer full-length protein, the decrease in mature miRNA expression varied only between 40 and 60% 48 h after the second round of transfection (Supplementary Figure S2B). (Similar results were obtained with the two different control siRNAs, namely siCt1 and siCt2, and with the two different siRNAs homologous to Dicer sequences, that is, si1Dcr and si2Dcr, data not shown.)

We next analysed Dicer mRNA and protein expression levels in 21 human breast cancer cell lines and in HMECs. We investigated the clinicopathological significance of Dicer mRNA expression in the 21 cell lines and in HMECs. As we have found for the human cancer progression cellular model, a very significant association was observed between the mRNA levels of Dicer and cell phenotype, with significantly lower levels in mesenchymal phenotype cells (*P*=0.0002, Table 1; Supplementary Figure S3). Epithelial and mesenchymal phenotypes were determined using phase-contrast microscopy and by examining E-cadherin and vimentin expression by western blotting (data not shown). Interestingly, Dicer mRNA expression was found to be tumour stage-dependent, with increasing levels between immortalised cells and cell lines established from ductal carcinoma *in situ*, and a subsequent marked decrease in cell lines established from invasive ductal carcinoma and in cell lines established from metastases (*P*-value <0.0001, Table 1).

To determine whether mRNA levels (a, b, and c variants) reflected full-length protein expression, 18 out of the 21 breast cancer cell lines and HMECs cells were examined for Dicer protein expression by western blotting. Semi-quantitative measures of protein level showed 72% of concordance between RNA and protein levels (Supplementary Figure S3). Overall, 74% of the cell lines showed a significant association with epithelial–mesenchymal phenotypes (Supplementary Figure S3).

By western blotting we detected two alternatively spliced isoforms (113.2 and 93 kDa) corresponding to the d and e variants, respectively (Figure 1 and http://www.abcam.com/index.html?pageconfig=reviews&intAbreviewID=4856&intAbID=14601). These variants were detectable in normal and immortalised cells, they were highly expressed in some breast cancer cell lines of the epithelial phenotype, and absent in the majority of the mesenchymal phenotype cell lines (data not shown).

### Evaluation of the clinical and prognostic significance of Dicer expression in breast cancer patients: association with distant metastases

We further examined the potential clinical significance of Dicer expression levels in breast cancer tissue samples obtained from two independent populations of breast carcinomas. The clinical characteristics of the two tumour populations are described in Supplementary Table S1. There was a clear association between Dicer mRNA expression and lymph node status; a significantly lower mRNA Dicer expression was observed in cases with lymph node metastases (N1: mean=7.316 *vs* N0: mean=12.240; *P*=0.0200, Supplementary Table S2). There was also a significant association of mRNA levels with luminal A breast cancer subtype. Dicer mRNA levels were lower in tumours that were not classified as luminal A cancer subtype (luminal B, HER2+, and basal like) (luminal A: mean=9. 487 *vs* non-luminal A: mean=6.126; *P*=0.0481, Supplementary Table S2).

**Figure 4:**
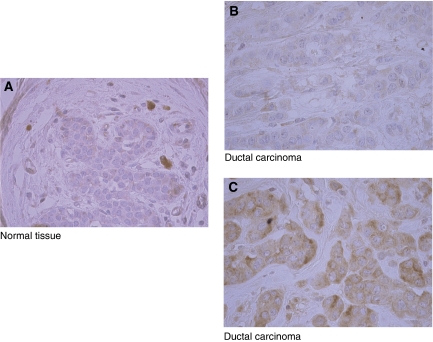
Immunohistochemical analysis of Dicer protein expression in normal and breast cancer tissues. (**A**) Low-intensity staining in the cytoplasm of epithelial luminal normal breast cells. Non-specific staining of mast cells. (**B**) Low-intensity staining in the cytoplasm of infiltrating ductal carcinomatous cells. (**C**) High-intensity staining in the cytoplasm of infiltrating ductal carcinomatous cells. Images are shown at magnification, × 40 (panels A–C).

In a preliminary IHC analysis with eight normal mammary gland tissues, Dicer protein was found to be expressed uniformly in the cytoplasm of the luminal cells (Figure 4A). The signal within the cytoplasm of luminal cells was weak (corresponding to intensity 1). No positive immunoreactivity was found either in basal or in stromal cells. In mammary tumour cells, Dicer showed variable expression patterns. Some tumours were negative for Dicer staining (data not shown), whereas other tumour tissues showed a staining intensity comparable with that found in normal tissues (intensity 1 and >60% of stained cells) (Figure 4B). Nevertheless, other tumours showed stronger cytoplasmic Dicer staining with a non-uniform pattern in a reduced tumour cell population (intensity 2 and ⩽60% of stained cells) (Figure 4C). We have found a significant inverse correlation between staining intensity and percentage of stained cells (*P*=0.013, Supplementary Table S3); therefore, either can be used as a staining score. The majority (67%) of tumours in TMA analysis (58 out of 86) was of staining intensity 1 and 62% (36 out of 58) of them showed more than 60% of stained cells compared with only 29% (5 out of 21) of tumours showing staining intensity scored as 2 (Supplementary Table S3).

We found a significant association between Dicer protein expression and hormone receptor status. The intensity 1 and no immunoreactivity were more frequently observed in ER- and PR-positive tumours (ER+ 71.8% *vs* ER− 46.7%, *P*=0.008; PR+ =71.9% *vs* PR− 54.6%, *P*=0.019, Supplementary Table S3). The population of tumours with negative staining (intensity 0) is very small (7 out of 86); therefore, in statistical analysis we grouped them with intensity 1 tumour population (Supplementary Table S3).

Dicer protein expression was also correlated with cancer subtype. Tumours of intensity 1 staining (identical to the intensity staining found in normal luminal cells) were correlated with luminal A cancer subtype (cancer subtype *P*=0.023; luminal A 71.9% *vs* non-luminal A 52.6%, *P*=0.0174, Supplementary Table S3).

**Figure 5:**
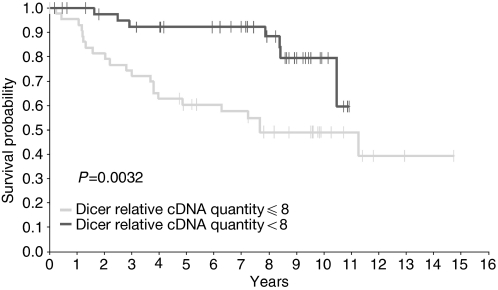
Kaplan–Meier estimates of metastatic-free survival by Dicer's expression among the mRNA population. 
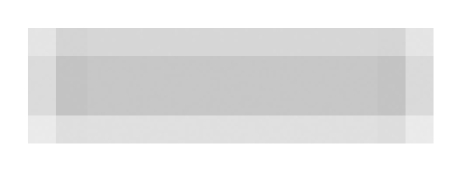
>8 cDNA relative quantity (7 out of 43 progressions; 8-year event-free survival probability 88.3% (95% CI: 77.3–99.3); 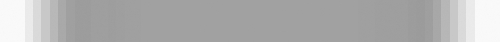
⩽8 cDNA relative quantity (22 out of 45 progressions; 8-year event-free survival probability 49.1% (95% CI: 33.6–64.6); log-rank test: *P*=0.0032.

Finally, we examined whether Dicer mRNA and protein expression could be useful in the prognosis of breast cancer patients. Only mRNA expression showed association with patient outcome (Figure 5). Protein levels were not predictive for patient's survival (data not shown). The probability of metastasis-free survival was significantly lower for patients with levels of Dicer mRNA <8 compared with those with levels >8 (Figure 5, HR: 3.36, *P*=0.0032). Interestingly, when an adjustment was made for each clinocopathological prognosis criteria, the HR values remained generally similar and for the most part, significantly >1 (Table 2).

## Discussion

The aim of this study was to evaluate independently the clinical relevance of RNA and the protein expression of Dicer in breast carcinoma samples. Furthermore, we investigated whether Dicer expression could vary during tumour and metastasis progression using human and mouse cellular models, as well as breast cancer cell lines.

We have found that Dicer mRNA expression was variable in breast carcinoma samples and that lower levels were more frequent in patients with metastatic relapse, indicating that Dicer mRNA levels are clinically relevant. Low mRNA levels were significantly associated with cancer subtypes other than luminal A (luminal B, HER2+, and basal like) and with metastases to lymph nodes, both of which are clinical parameters of aggressiveness. Interestingly, we have shown that Dicer expression appeared downregulated in two independent metastatic bone derivative clones of a breast cancer cell line. Transcriptomic analyses that were carried out in MDA-MB-231 parental and in BO2 clone have shown the same decrease in Dicer expression (Bellahcène *et al*, 2007; Garcia *et al*, 2008) (R Bachelier and P Clézardin, personal communication).

In *Dicer*-conditional knockout mice, the loss of Dicer resulted in increased DNA damage (Mudhasani *et al*, 2008), and the timing of locus replication during S-phase has been shown to be very sensitive to the influence of Dicer hemi-depletion (Jørgensen *et al*, 2007). The study by Kumar *et al*, 2007 nicely showed that defective miRNA processing machinery improves the transformation capacities of cancer cells. Dicer expression has been previously investigated in other tumour types and in breast cancers. Our RT–PCR results are consistent with those of Blenkiron *et al* (2007) who have found by microarray analysis low expression of Dicer in luminal B, HER2+, and basal-like tissues. A relationship between high Dicer mRNA levels analysed by microarray and increased disease-free survival was found in two different cohorts of patients suffering from breast cancer (Merritt *et al*, 2008). Findings in other tumour types showed decreased or increased Dicer expression associated with aggressive cancers, evoking a tissue specificity of Dicer deregulation expression in cancers. High Dicer expression was found in prostate and in oesophageal carcinomas, whereas downregulation was found in advanced lung adenocarcinoma (Chiosea *et al*, 2006, 2007; Sugito *et al*, 2006). It is noteworthy that the *Dicer* gene is located at the subtelomeric region 14q32.13, which carries a cluster of imprinted genes critically affected by various deletions, rearrangements, and epimutations that might potentially influence the methylation status of this region during tumour progression (Kagami *et al*, 2008). However, it has also been shown that the *Dicer* gene was not methylated in lung cancers (Karube *et al*, 2005), and that treatment of cells with a histone deacetylase inhibitor does not change levels of Dicer mRNA, with minimally altered Dicer message levels (Wiesen and Tomasi, 2009). Abnormalities in the copy number of the *Dicer* gene were found in breast and ovarian cancers, as well as in melanoma (Zhang *et al*, 2006). Two studies in ovarian carcinomas appeared to be contradictive on Dicer expression, showing in tumours of epithelial type higher or lower Dicer expression both associated with poor prognosis (Flavin *et al*, 2008; Merritt *et al*, 2008). Merritt *et al.* showed a significant association of Dicer mRNA levels with survival, whereas Flavin *et al.* failed to show an association with survival on the basis of protein expression. However, it is unclear which Dicer isoforms were analysed in the two studies. Contrary to other organisms, mammals have a single *Dicer* gene but its expression is a highly regulated process with 11 alternatively spliced variants and 3 full-length forms (http://www.ncbi.nlm.nih.gov/ieb/research/acembly/). Characterisation of the 5′UTR of *Dicer* has defined three non-coding exon 1 variants as well as several alternatively spliced non-coding 5′exons, and the 3 full-length forms show differences in 3′UTR sequence (Irvin-Wilson and Chaudhuri, 2005; Singh *et al*, 2005). Some spliced Dicer mRNAs putatively encode transcribed proteins as we have shown by western blotting that the two splice variants, d and e, were highly expressed in some breast cancer cell lines, whereas totally absent in others in addition to being detectable in normal and immortalised breast cells. These variants showed 94% homology with a long-form 5′UTR variant expressed in breast cells, whereas no homology was found with a short-form 5′UTR variant also expressed in breast cells (Irvin-Wilson and Chaudhuri, 2005). The variants d and e were not only expressed in breast tissues but were also detected in HeLa cells (data not shown). We may ask whether the d and e isoforms could be functional as they both contain the ribonuclease III domain, the dsRNA binding domain while only the d isoform contains a PAZ domain. It has been shown previously that Dicer functions both as a monomer and a dimer (Carmell and Hannon, 2004). Given that the e variant contains two ribonuclease domains, we expect it to function as a monomer and the d variant with one ribonuclease domain to function as a dimer. In our study, these variants were not targeted by siRNA in HeLa cells, and the inactivation of full-length a, b, and c forms did not lead to a complete elimination of mature miRNAs. One of the miRNA species showed only 40% decreased expression; however, we cannot exclude that some mature miRNAs may have long turnover times and therefore the miRNA observed is residual before the knock down of Dicer.

Regulation of Dicer expression seems largely post-transcriptional as cellular Dicer mRNA measured by PCR is not well correlated with protein expression (Wiesen and Tomasi, 2009). The two previous studies on Dicer expression in breast cancers failed to compare RNA and protein expression (Blenkiron *et al*, 2007; Merritt *et al*, 2008). In breast cancer cell lines, we have found 72% concordance between mRNA and protein levels. A high correlation between protein and mRNA levels was found only for breast cancer cell lines of the mesenchymal phenotype. Although Dicer mRNA levels appeared to be predictive for metastasis-free survival, protein expression was not informative for survival. Furthermore, we obtained conflicting data between mRNA and protein expression regarding association with clinical parameters. Higher mRNA levels were correlated with luminal A cancer subtype, whereas negative or weak immunoreactivty in the TMA study were associated with this cancer subtype. It is noteworthy that low immunoreactivity has the same level of staining observed in normal breast tissues.

The Dicer protein is a part of the RISC loading, small RNA processing complex, including its co-factor TRBP (TAR RNA binding protein), PACT, and Ago2, which could affect its stability depending on their respective expression in cancer cells (Chiosea *et al*, 2006, 2007; Lee *et al*, 2006; Zhang *et al*, 2006; Blenkiron *et al*, 2007). Recent publications highlight the intricate complexity of Dicer expression regulation showing that Dicer is a high probability target of multiple miRNAs. Dicer is believed to be a ‘hub’ through a novel regulatory loop in which the mature miRNA (primarily let-7) processed by Dicer affects Dicer expression at both protein and mRNA levels (Johnson *et al*, 2007; Asirvatham *et al*, 2008; Forman *et al*, 2008; Roush and Slack, 2008; Selbach *et al*, 2008; Tokumaru *et al*, 2008). let-7 might confer varying degrees of Dicer translational inhibition versus mRNA instability depending on specific target sites. Recognition sites for miRNAs have been reported to be mainly located at the 3′UTR of transcripts. The Dicer RNA variants a and b contain a very long 3′UTR, whereas c, d, and e variants contain very short 3′UTR (Figure 1). Multiple let-7-binding sites were found in Dicer 3′UTR (Forman *et al*, 2008). Recently, let-7 target sites were also found in the Dicer coding region, and it was suggested that sites in the coding region and in 3′UTR may differ in mechanism (Forman *et al*, 2008; Selbach *et al*, 2008). Multiplicity of miRNA-binding sites in the same 3′UTR exerts a stronger effect on protein production than on mRNA levels (Selbach *et al*, 2008). It is noteworthy that binding of miRNA in the 3′UTR has only mild effects on translation. In the coding region, a greater number of nucleotides which bind to miRNA, suggest that these sites may activate mRNA degradation similar to that in *Arabidopsis* (Xie *et al*, 2003). let-7 miRNAs are largely expressed in many tissues, including the mammary gland (Landgraf *et al*, 2007). Human *let-7* genes map to regions altered or deleted in human tumours, and the majority of let-7 family members is considered as tumour suppressor miRNAs, with decreased expression in different tumour types, such as serous ovarian carcinomas and lung tumours (Takamizawa *et al*, 2004; Johnson *et al*, 2007; Nam *et al*, 2008). We can hypothesise that in normal and immortalised mammary cells and in breast tumours with good prognosis (luminal A cancer subtype) let-7 miRNAs repress the translation of Dicer variants with long 3′UTR, whereas they induce the down-expression of the short 3′UTR variants. In some cancer cells, let-7 downregulation may render the long 3′UTR variants accessible to other miRNAs or RNA binding proteins that affect mRNA stability and thus may lead to a switch in the variant's downregulation (Selbach *et al*, 2008). In other cancer cells, expression of some miRNAs may lead to the degradation of all variants. In HMLER cells and in mesenchymal phenotype cancer cell lines, we have found both decreased expression of mRNA and of the three protein forms (full length, d and e spliced forms). We cannot rule out that alterations in the *Dicer* gene or in *Dicer* gene expression may exist in these cells. Breast tumours of the mesenchymal phenotype are metaplastic carcinomas that are rare in clinical practice. Recently, it has been shown that metaplastic carcinomas represent a subtype of basal-like cancers (Weigelt *et al*, 2008). However, the mesenchymal phenotype may not be a prerequisite for tumour cells to become metastases. The cells expressing hTERT, LT, and H-rasV12 (HMLER) form colonies in soft agar and tumours in nude mice (Elenbaas *et al*, 2001). No signs of metastatic spread are observed in tumour-bearing mice. The mesenchymal phenotype is not correlated with the capacity of the cells to metastasise in the 67 NR mouse model (Lou *et al*, 2008). Lou *et al* recently showed that 67NR and 66c14 sub-lines exhibit morphological characteristics of the mesenchymal phenotype. 168FARN are a mixed population with predominance of the mesenchymal population. Finally, the metastatic sub-lines, 4T07 and 4T1 exhibit epithelial characteristics. Cells of the mesenchymal phenotype express less Dicer protein than those of the epithelial phenotype, but more specifically there was an inverse correlation between Dicer and vimentin protein levels in the sub-lines. 4T1 cells (E) showed high levels of E-cadherin and weak expression of vimentin, 66c14 (M) expressed no E-cadherin but vimentin with the same levels found in 4T1 cells, and 67NR (M) did not express E-cadherin but vimentin at high levels (Lou *et al*, 2008).

In conclusion, Dicer mRNA expression is significantly correlated with the occurrence of distant metastases, even after adjusting for other prognostic parameters. We found that Dicer mRNA expression had an independent prognostic value on metastatic disease in breast cancers. Our findings suggest that the downregulation of Dicer expression may be related to aggressiveness and metastatic spread of tumours. Thus, we propose that Dicer mRNA be considered as a novel predictive biomarker in breast cancer metastatic disease. Further definition of the variant(s) responsible for the association between RNA expression levels and metastatis-free survival (i.e., between variants a, b, and c) could also increase the power of the marker in other types of cancer.

## Figures and Tables

**Table 1:** Quantitative RT–PCR analysis of *Dicer* expression in breast cancer cell lines and correlation with clinicopathological characteristics

**Biological and clinical**	**Cases**	**Measures**	**Dicer**	
**characteristics**	**n=23**	**111**	**Mean CI**	***P*-value**
*Cell phenotype*				
Epithelial	11	55	4.056 (3.230–5.093)	0.0002
Mesenchymal	7	35	1.763 (1.363–2.282)	
				
*Tumour stage*				
Immortalized cell	3	15	2.198 (1.577–3.064)	<0.0001
Intra-ductal carcinoma (DCIS)	4	20	4.107 (3.445–4.895)	
Invasive ductal carcinoma (IDC)	12	60	1.524 (NA)	
Metastases	1	5	1.369 (1.045–1.794)	

CI=confidence interval; RT–PCR=reverse transcription PCR.

**Table 2:** Metastasis free survival—comparison of the stratified hazard ratios

	**Metastatic progression**			
	**TMA population**		**mRNA population**	
**Subgroups**	**Metaprog/N**	**HR (95% CI) (2 vs 0/1)**	**Metaprog/N**	**HR (95% CI) (<=8 vs >8)**
Overall	13/79	1.25 (0.38–4.05)	29/87	3.36 (1.43–7.92)
Adjusted effect on age	13/79	1.38 (0.42–4.51)^a^	29/85	3.60 (1.53–8.47)
Adjusted effect on menopause	13/79	1.19 (0.37–3.88)	29/86	3.24 (1.37–7.67)
Adjusted effect on pT	13/79	1.77 (0.53–5.93)	29/86	3.23 (1.36–7.65)
Adjusted effect on histological type	13/79	1.05 (0.32–3.42)^a^	29/86	2.99 (1.27–7.05)
Adjusted effect on SBR	13/79	0.96 (0.29–3.17)	28/81	3.14 (1.32–7.45)
Adjusted effect on N	13/79	1.19 (0.36–3.93)	29/87	2.77 (1.17–6.52)
Adjusted effect on ER (%)	13/79	0.84 (0.23–3.03)	28/84	3.14 (1.32–7.47)
Adjusted effect on PR (%)	13/79	0.90 (0.26–3.14)	29/86	3.23 (1.37–7.62)
Adjusted effect on HER2	13/79	1.34 (0.40–4.48)	21/64	2.26 (0.85–6.00)
Adjusted effect on luminal A	12/77	1.07 (0.30–3.83)	21/63	2.64 (0.99–7.00)
Adjusted effect on cancer subtype	12/77	0.76 (0.18–3.18)	21/63	2.59 (0.97–6.89)

CI=confidence interval; ER=estrogen receptor; HR=hazard ratio; HER2=human epidermal growth factor receptor 2; N=lymph node status; PR=progesterone receptor; SBR=Scarff-Bloom-Richardson; TMA=tissue microarray.

If HR>1, the patient has a higher risk to have a metastatic reccurrence at time *t* compared with the reference item.

If HR <1, patient has a lower risk to have a metastatic reccurrence at time *t* compared with the reference item.

a Adjusting parameter statistically significant in the model.
