# Supplementary figures and images for: Prognostic value of Dicer expression in human breast cancers and association with the mesenchymal phenotype
Source: Br J Cancer. 2009 Aug 11;101(4):673–83. doi: 10.1038/sj.bjc.6605193 (PMC2736830; doi:10.1038/sj.bjc.6605193)

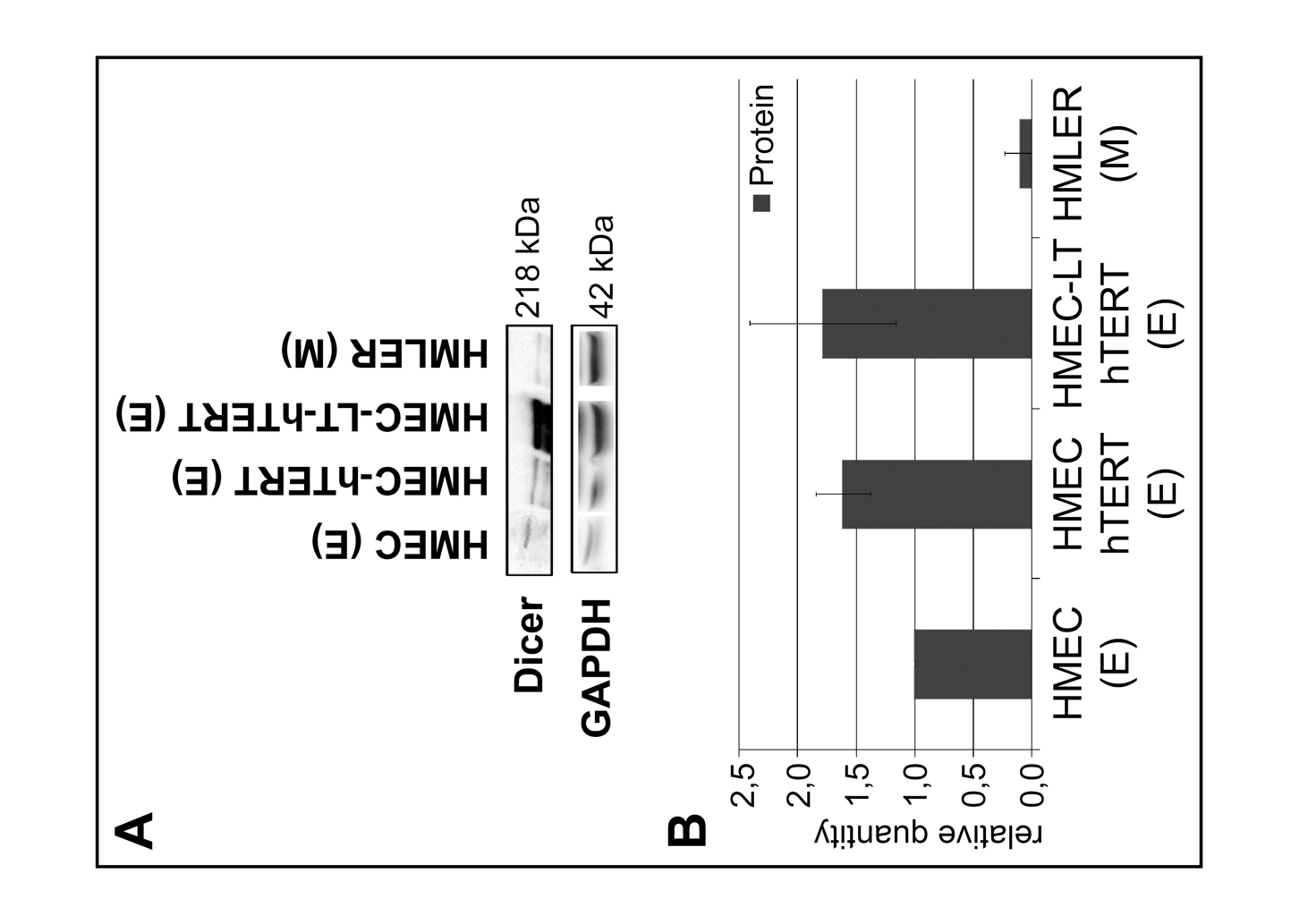

Supplement: Supplementary Figure S1 [file 6605193x1.jpg]

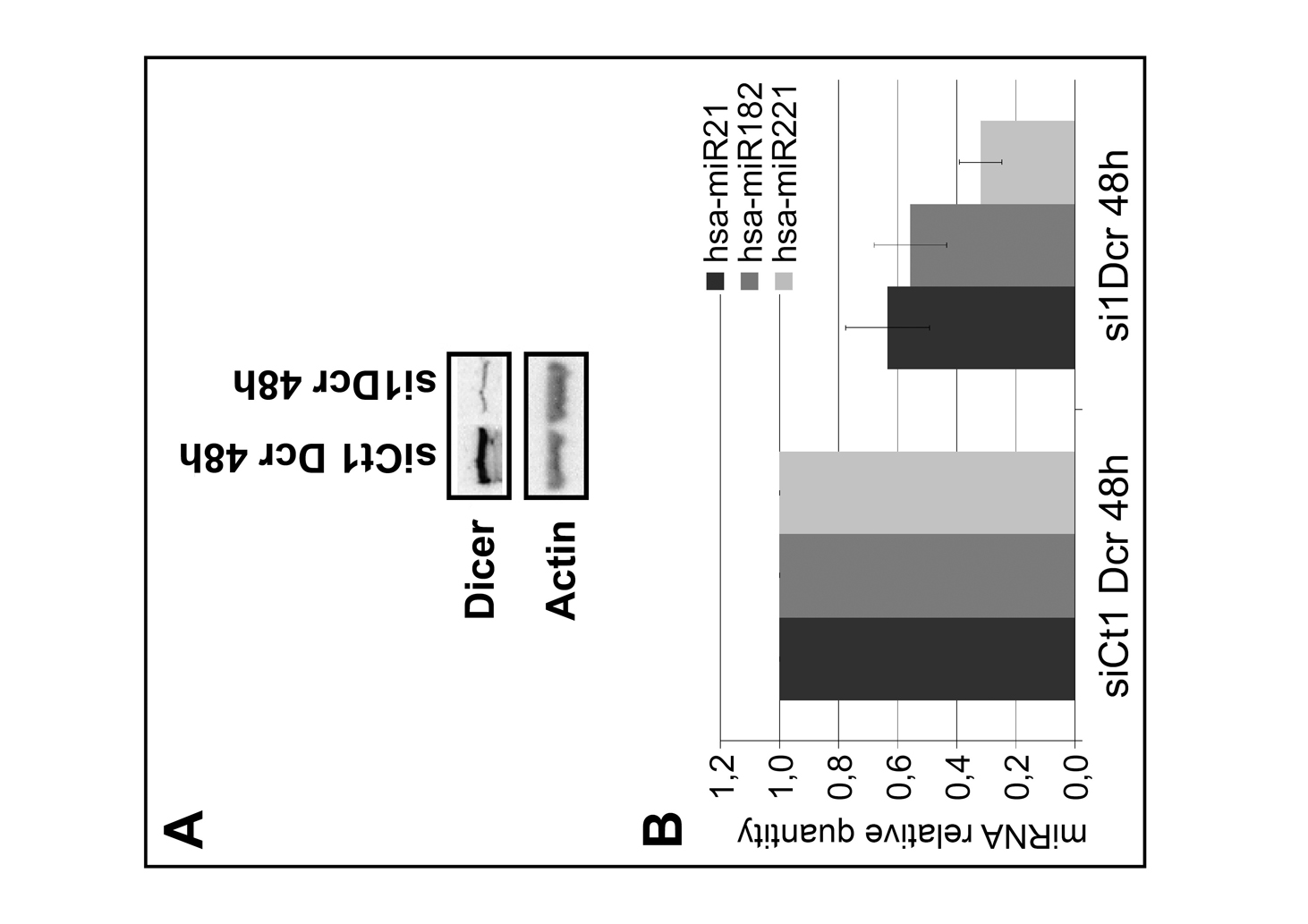

Supplement: Supplementary Figure S2 [file 6605193x2.jpg]

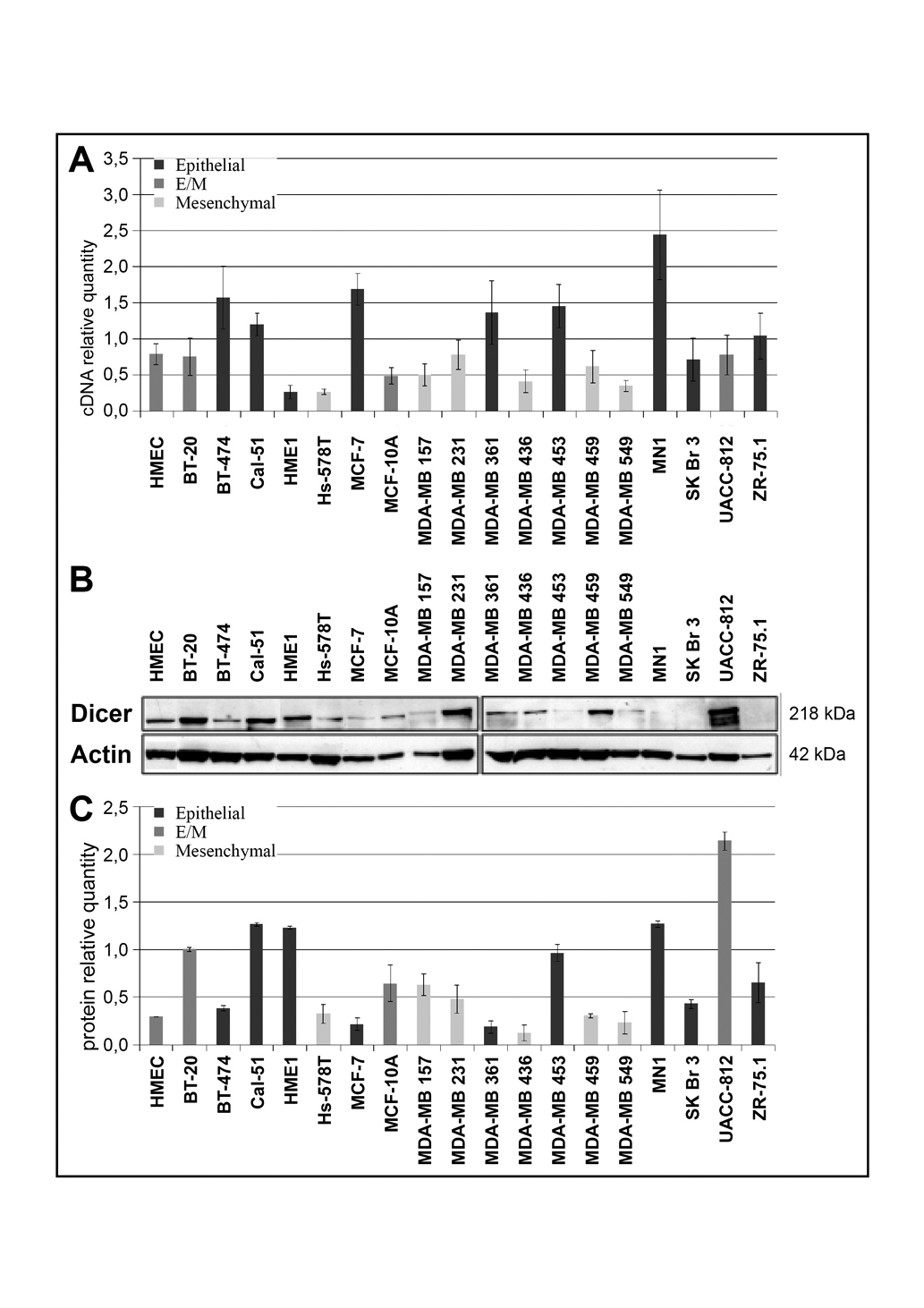

Supplement: Supplementary Figure S3 [file 6605193x3.jpg]
