# Supplementary material for: Prognostic value of Dicer expression in human breast cancers and association with the mesenchymal phenotype
Source: Br J Cancer. 2009 Aug 11;101(4):673–83. doi: 10.1038/sj.bjc.6605193 (PMC2736830; doi:10.1038/sj.bjc.6605193)
Supplement: Supplementary Table S1 [file 6605193x4.doc]

**Table S1: Comparison of TMA and mRNA population**

| **Variables** | **TMA population**  **N=86** | **mRNA population**  **N=104** |
| --- | --- | --- |
| Age |  |  |
| <50 years | 24 (27.9%) | 32 (31.4%) |
| ≥50 years | 62 (72.1%) | 70 (68.6%) |
| *Missing* | *0* | *2* |
| Menospausal status |  |  |
| Yes | 55 (67.9%) | 65 (64.4%) |
| No | 26 (32.1%) | 36 (35.6%) |
| *Missing + hysterectomy* | *5* | *3* |
| pT |  |  |
| ≤20 mm | 42 (48.8%) | 14 (13.9%) |
| ]20-50] mm | 39 (45.3%) | 56 (55.4%) |
| >50 mm | 5 (5.8%) | 31 (30.7%) |
| *Missing* | *0* | *3* |
| Histological type |  |  |
| Ductal | 71 (82.6%) | 94 (91.3%) |
| Lobular | 7 (8.1%) | 4 (3.9%) |
| Mixed | 2 (2.3%) | 4 (3.9%) |
| Other | 6 (7.0%) | 1 (1.0%) |
| *Missing* | *0* | *1* |
| Histological grade (SBR) |  |  |
| 1 | 14 (16.3%) | 12 (12.4%) |
| 2 | 36 (41.9%) | 42 (43.3%) |
| 3 | 36 (41.9%) | 43 (44.3%) |
| *Missing* | *0* | *7* |
| Invaded nodes |  |  |
| N0 | 65 (75.6%) | 18 (17.3%) |
| N1 | 21 (24.4%) | 86 (82.7%) |
| Estrogen Receptor |  |  |
| - | 15 (17.4%) | 21 (21.0%) |
| + | 71 (82.6%) | 79 (79.0%) |
| *Missing* | *0* | *4* |
| Progesterone Receptor |  |  |
| - | 22 (25.6%) | 25 (24.5%) |
| + | 64 (74.4%) | 77 (75.5%) |
| *Missing* | *0* | *2* |
| HER2 status |  |  |
| - | 78 (94.0%) | 60 (77.9%) |
| + | 5 (6.0%) | 17 (22.1%) |
| *Missing* | *3* | *27* |
| Basal-like |  |  |
| Yes | 14 (16.3%) | 8 (10.5%) |
| No | 72 (83.7%) | 68 (89.5%) |
| *Missing* | *0* | *28* |
|  |  |  |
| Cancer subtype |  |  |
| Luminal A | 64 (77.1%) | 51 (67.1%) |
| Luminal B | 4 (4.8%) | 16 (21.1%) |
| Basal-like | 14 (16.9%) | 8 (10.5%) |
| HER2+ | 1 (1.2%) | 1 (1.3%) |
| *Missing* | *3* | *28* |
| Metastasis at diagnosis |  |  |
| Yes | 7 (8.1%) | 15 (14.6%) |
| No | 79 (91.9%) | 88 (85.4%) |
| *Missing* | *0* | *1* |
