# Supplementary material for: Prognostic value of Dicer expression in human breast cancers and association with the mesenchymal phenotype
Source: Br J Cancer. 2009 Aug 11;101(4):673–83. doi: 10.1038/sj.bjc.6605193 (PMC2736830; doi:10.1038/sj.bjc.6605193)
Supplement: Supplementary Table S2 [file 6605193x5.doc]

| **Table S2** Quantitative RT-PCR analysis of *dicer* expression in breast cancer biopsies and correlation with clinicopathological characteristics | | | | |
| --- | --- | --- | --- | --- |
| *Tumour Characteristics* | *Cases* | *Measures* | *dicer* | |
|  | *n=104* | *n=374* | *Mean (95%IC)* | *P-value* |
| *Tissue type* |  |  |  |  |
| normal | *3* | *12* | 3.728 (NA) | 0.2734 |
| tumour | *104* | *374* | 7.980 (6.832-9.322) |  |
| *Menopausal status* |  |  |  |  |
| yes | *65* | *233* | 8.883 (7.237-10.903) | 0.0797 |
| no | *36* | *131* | 6.598 (5.161-8.435) |  |
| *Histological grade (SBR)* |  |  |  |  |
| 1 | *12* | *37* | 7.110 (4.670-10.827) | 0.6177 |
| 2 | *42* | *147* | 8.647 (6.713-11.139) |  |
| 3 | *43* | *170* | 7.527 (5.907-9.592) |  |
| *Tumour size* |  |  |  |  |
| < 20 mm | *14* | *51* | 6.741 (4.394-10.343) | 0.6823 |
| [20-50] mm | *56* | *197* | 7.668 (6.248-9.410) |  |
| >50 mm | *31* | *118* | 8.979 (6.621-12.176) |  |
|  |  |  |  |  |
| *Histological type* |  |  |  |  |
| ductal | *94* | *344* | 7.584 (6.448-8.920) | 0.2135 |
| lobular | *4* | *16* | 13.895 (NA) |  |
| other | *5* | *13* | 10.409 (NA) |  |
| *N status* |  |  |  |  |
| N1 | *86* | *320* | 7.316 (6.219-8.606) | 0.0200 |
| N0 | *18* | *54* | 12.240 (8.152-18.376) |  |
| *Metastasis at diagnosis* |  |  |  |  |
| yes | *15* | *36* | 10.629 (7.451-15.163) | 0.1545 |
| no | *88* | *332* | 7.572 (6.394-8.966) |  |
| *Estrogen Receptor* |  |  |  |  |
| ER+ (≥ 10%) | 79 | 284 | 8.417 (6.94-10.205) | 0.1002 |
| ER- (< 10%) | 21 | 78 | 6.094 (4.842-7.669) |  |
| *Progesterone Receptor* |  |  |  |  |
| PR+ (≥ 10%) | 77 | 273 | 7.750 (6.468-9.285) | 0.7862 |
| PR- (< 10%) | 25 | 95 | 8.182 (5.966-11.223) |  |
| *HER2 status* |  |  |  |  |
| - | *60* | *196* | 8.542 (6.697-10.897) | 0.3492 |
| + | *17* | *67* | 6.520 (4.693-9.057) |  |
| *Cancer subtype* |  |  |  |  |
| Luminal A | *51* | *164* | 9.487 (7.287-12.352) | 0.2200 |
| Luminal B | *16* | *65* | 6.571 (4.666-9.253) |  |
| HER2 + | *1* | *2* | 2.490 (NA) |  |
| Basal-like | *8* | *27* | 5.218 (3.399-8.010) |  |
| *Luminal A* |  |  |  |  |
| Yes | *51* | *164* | 9.487 (7.287-12.352) | 0.0481 |
| No | *25* | *94* | 6.126 (4.714-7.962) |  |
