# Supplementary material for: Prognostic value of Dicer expression in human breast cancers and association with the mesenchymal phenotype
Source: Br J Cancer. 2009 Aug 11;101(4):673–83. doi: 10.1038/sj.bjc.6605193 (PMC2736830; doi:10.1038/sj.bjc.6605193)
Supplement: Supplementary Table S3 [file 6605193x6.doc]

| **Table S3** Association between clinical data and % of Dicer marked cells among the TMA population | | | | |
| --- | --- | --- | --- | --- |
| Mean ± SD (median [min,max]  **(%)** | **Intensity 0**  **(n=7)** | **Intensity 1**  **(n=58)** | **Intensity 2**  **(n=21)** | **P-value**  **0/1 vs 2** |
| Age  < 50 years  ≥ 50 years | 0 (0.0%)  7 (11.3%) | 17 (70.8%)  41 (66.1%) | 7 (29.2%)  14 (22.6%) | 0.580 |
| Menopausal status :  Yes  No | 7 (12.7%)  0 (0.0%) | 34 (61.8%)  20 (76.9%) | 14 (25.5%)  6 (23.1%) | 1.000 |
| pT  <= 20 mm  [20 – 50] mm  > 50 mm | 3 (7.1%)  3 (7.7%)  1 (20.0%) | 26 (61.9%)  29 (74.4%)  3 (60.0%) | 13 (31.0%)  7 (18.0%)  1 (20.0%) | 0.361 |
| Histological type:  Ductal  Lobular  Mixed  Others | 4 (5.6%)  3 (42.9%)  0 (0.0%)  0 (0.0%) | 48 (67.6%)  4 (57.1%)  2 (100.0%)  4 (66.7%) | 19 (26.8%)  0 (0.0%)  0 (0.0%)  2 (33.3%) | 0.347 |
| Histological grade (SBR):  1  2  3 | 1(7.1%)  4 (11.1%)  2 (5.6%) | 10(71.4%)  26 (72.2%)  22 (61.1%) | 3 (21.4%)  6 (16.7%)  12 (33.3%) | 0.266 |
| N status :  N0  N1 | 5 (7.7%)  2 (9.5%) | 46 (70.8%)  12 (57.1%) | 14 (21.5%)  7 (33.3%) | 0.381 |
| M status :  M0  M1 | 6 (7.6%)  1 (14.3%) | 52 (65.8%)  6 (85.7%) | 21 (26.6%)  0 (1.1%) | 0.187 |
| Estrogen receptor :  % marked cells (count):  ER- (< 10%)  ER+ (≥ 10%) | 0 (0.0%)  7 (9.9%) | 7 (46.7%)  51 (71.8%) | 8 (53.3%  13 (18.3%) | 0.008 |
| Progesterone receptor :  % marked cells (count)  PR- (< 10%)  PR+ (≥ 10%) | 0 (0.0%)  7 (10.9%) | 12 (54.6%)  46 (71.9%) | 10 (45.5%)  11 (17.2%) | 0.019 |
| HER2 status  -  + | 6 (7.7%)  0 (0.0%) | 53 (68.0%)  3 (60.0%) | 19 (24.4%)  2 (40.0%) | 0.597 |
| Luminal A  Yes  No | 6 (9.4%)  0 (0.0%) | 46 (71.9%)  10 (52.6%) | 12 (188%)  9 (47.4%) | 0.0174 |
| Cancer subtype  Luminal A  Luminal B  Basal-like  HER2+ | 6 (9.4%)  0 (0.0%)  0 (0.0%)  0 (0.0%) | 46 (71.9%)  3 (75.0%)  0 (0.0%)  7 (50.0%) | 12 (188%)  1 (25.0%)  1 (100.0%)  7 (50.0%) | 0.023 |
| Dicer  % marked cells (count)  ≤ 60%  > 60% | 7 (15.6%)  0 (0.0%) | 22 (48.9%)  36 (87.8%) | 16 (35.6%) 5 (12.2%) | 0.013 |
