# Supplementary material for: Prognostic value of Dicer expression in human breast cancers and association with the mesenchymal phenotype
Source: Br J Cancer. 2009 Aug 11;101(4):673–83. doi: 10.1038/sj.bjc.6605193 (PMC2736830; doi:10.1038/sj.bjc.6605193)
Supplement: Supplementary Information [file 6605193x7.doc]

Supplementary Materials and Methods

*Steroid receptor assay*

ER and PR status were carried out by established immunohistochemical (IHC) method of avidin-biotin-peroxidase complex (Vectastain Elite ABC reagent Vector, Abcys, Paris, France) described previously (Tang *et al.,* 1997) using a mouse monoclonal anti-ERα antibody (clone 1D5, Dako, Trappes, France) and a mouse monoclonal anti-PRA (clone PR 636, Dako, Trappes, France). As cut-off point for positivity ≥ 10% frequency of positive stained tumor cells was used.

*Primers sequences and real time PCR experimental procedures*

Dicer specific primer pair sequences used in RT-PCR amplification was: forward 5'TTAAC- CTTTTGGTGTTTGATGAGTGT and reverse 5'GCGAGGACATGATGGACAATT. This primers set allow to detect the three full length variants a, b and c (National Center for Biotechnology Information, <http://www.ncbi.nlm.nih.gov/IEB/Research/Acembly/>). Information about alternatively spliced Dicer variants was obtained from Aceview **(**http://aceview.com/).Cyclophilin B (CPB), β-actin and phosphoglycerokinase (PGK) were used as reference genes for the results normalisation. The primer pairs and probes were designed with Universal Probe Library website Primers of each pair were located in different exons to avoid genomic amplification. All reactions were performed using LightCycler FastStart DNA Master PLUS SYBR Green I kit for Dicer or LightCycler Taqman Master for reference genes (Roche Applied Science, Basel, Switzerland). The reaction mixture contained FastStart TaqDNA polymerase, reaction buffer, deoxynucleoside triphosphate mixture, MgCl2, 0.25 μM of each primer, and 1.67 μl (5 μL for reference genes) of a 1/60 dilution of first-strand cDNA from cells. The final reaction volume was 6.67 μl (10 μL for reference genes). Thermocycling conditions for dicer gene were designed in four consecutive steps (the Roche Applied Science Universal Probe Library’s thermocycling conditions were used for the reference genes). Step 1: denaturation and polymerase activation by 10 min heating at 95°C prior amplification. Step 2 (45 cycles): denaturation for 10 sec at 95°C, annealing for 10 sec at 55°C and extension for 6 sec at 72°C. Step 3: melting which consisted of incrementing the temperature from 45°C to 95°C by steps of 0.1°C. Step 4: cooling for 30 sec at 40°C for one cycle. This melting curve program was used to check the specificity of each dicer RT-PCR. All standards or samples were analyzed in duplicate and each experiment was repeated at least three times. All fluorescence data were analyzed by the LightCycler 4.0 software (Roche Applied Science, Basel, Switzerland) and Ct results were exported to Excel sheets.

A large amount of cDNA was prepared from the HME-1 cell line prior to the experiment. It was then diluted, aliquoted and used as a calibrator for all RT-PCR runs. For relative quantification and normalization, the comparative Ct (or Eff-ΔΔC) method was used.

***Breast Cancer cell lines***

The following 21 breast cancer cell lines were obtained from the American Type Culture Collection ([http://www.ATCC.org](http://www.ATCC.org/)): BT20, BT474, Cal51, Cama1, HBL100, HCC1937, HS578T, MCF10A, MCF12A, MCF7, MDAMB157, MDAMB231, MDAMB361, MDAMB436, MDAMB453, MDAMB459, MDAMB549, SKBr3, T47D, UACC812, ZR75.1. Cells grown according to American Type Culture Collection protocols to 90% confluency were collected for total RNA and protein preparation. HMECs and HME-1 (Lonza, Basel, Switzerland) were cultured in MEGM at 37°C with CO2 as described (Stampfer *et al.,* 1980). Breast cancer cell lines clinical and biological characteristics were described in Neve et al. (Neve *et al.,* 2006).

Supplementary figure legends

**Figure S1:**

**Expression of Dicer in the human breast tumour progression cellular-model relative to GAPDH**

1. Western blot analysis of Dicer expression in the four human breast cell lines HMEC, HMEC+ hTERT, HMEC+LT+hTERT and HMLER (expressing H-rasV12) cells. The signal intensity of Dicer was normalized to that of GAPDH. Rabbit polyclonal antibodies directed against GAPDH were used (Sigma-Aldrich, Saint Louis, MO).
2. Dicer protein levels were quantified using Quantity One software (BioRad, Marnes-la-Coquette, France) and expressed as protein relative quantity. The ratios of Dicer/GAPDH of three independent studies were expressed as mean ± SD.

**(E)** means epithelial phenotype **(M)** means mesenchymal phenotype

**Figure S2**:

**Expression of mature miRNA in Dicer knocked-down human tumour cells**

1. Western blot analysis of Dicer expression in HeLa cells 48 hrs after the second round of transfection by the siRNA directed against Dicer si1Dcr or the corresponding nontargeting siRNA si-Ct1. The signal intensity of Dicer was normalized to that of actin.
2. Expression of hsa-miR-21 ( ), hsa-miR-182 ( ) and hsa-miR-221 ( ) were measured by real-time RT-PCR in HeLa cells transfected by si-Ct1 or si1Dcr. Cells were harvested 48 hrs after the second round of transfection. Reactions were performed in triplicate and normalized to RNU44 cycle threshold values.

**Figure S3:**

**Comparative analyses of Dicer expression in normal HMECs and in breast cancer cell lines**

1. Total RNA was extracted and Dicer mRNA relative levels were studied by real-time RT-PCR, and each bar represents the mean ± SD of the PCRs in triplicate.
2. Western blot analysis was done to investigate Dicer protein levels. The signal intensity of Dicer was normalized to that of actin.
3. Dicer protein levels were quantified using Quantity One software (BioRad, Marnes-la-Coquette, France) and expressed as protein relative quantity. The ratios of Dicer/actin of three independent studies were expressed as mean ± SD.
